# Cesium Toxicity Alters MicroRNA Processing and AGO1 Expressions in *Arabidopsis thaliana*

**DOI:** 10.1371/journal.pone.0125514

**Published:** 2015-05-06

**Authors:** Il Lae Jung, Moonyoung Ryu, Seok Keun Cho, Pratik Shah, Ju Hye Lee, Hansol Bae, In Gyu Kim, Seong Wook Yang

**Affiliations:** 1 Department of Radiation Biology, Environmental Radiation Research Group, Korea Atomic Energy Research Institute, Daejeon, 305–353, Republic of Korea; 2 Department of Plant and Environmental Sciences, Faculty of Science, University of Copenhagen, Thorvaldsensvej 40, DK-1871, Frederiksberg, Copenhagen, Denmark; East Carolina University, UNITED STATES

## Abstract

MicroRNAs (miRNAs) are short RNA fragments that play important roles in controlled gene silencing, thus regulating many biological processes in plants. Recent studies have indicated that plants modulate miRNAs to sustain their survival in response to a variety of environmental stimuli, such as biotic stresses, cold, drought, nutritional starvation, and toxic heavy metals. Cesium and radio-cesium contaminations have arisen as serious problems that both impede plant growth and enter the food chain through contaminated plants. Many studies have been performed to define plant responses against cesium intoxication. However, the complete profile of miRNAs in plants during cesium intoxication has not been established. Here we show the differential expression of the miRNAs that are mostly down-regulated during cesium intoxication. Furthermore, we found that cesium toxicity disrupts both the processing of pri-miRNAs and AGONOUTE 1 (AGO1)-mediated gene silencing. AGO 1 seems to be especially destabilized by cesium toxicity, possibly through a proteolytic regulatory pathway. Our study presents a comprehensive profile of cesium-responsive miRNAs, which is distinct from that of potassium, and suggests two possible mechanisms underlying the cesium toxicity on miRNA metabolism.

## Introduction

Cesium is an alkali metal that exhibits similar physical and chemical properties to those of potassium. In contrast to potassium, an element essential to the development and growth of plants, the severe phytotoxicity of cesium has been suggested to be either caused by the element’s hindrance of potassium uptake, or by its competition for intracellular potassium binding sites. This leads to potassium starvation, which disrupts cellular activities [1, 2]. Because cesium is a relatively rare element in natural soil, the phytotoxicity of cesium barely bring on public concerns. However, soil pollution by cesium loading has been increasing in areas containing pollucite ore and factories manufacturing cesium-based commodities [3]. Even more serious soil contamination is caused by radioactive cesium. Two radioisotopes of cesium (^134^Cs and ^137^Cs) are well-defined environmental concerns, because of their harmful radioactivity and relatively long half-lives (30.1 years). Additionally, they are rapidly incorporated into biological systems [4, 5]. These isotopes are mostly produced by the testing of nuclear weapons, and unintentional or accidental exudations from nuclear power installations such as occurred during the Chernobyl and Fukushima disasters. Therefore, global concerns about the pernicious influence of cesium on plants have been heightened, especially with respects to ecological disturbance, crop productivity, food safety, and subsequently, human health.

Many studies have reported on the mechanisms by which cesium harms the development and growth of plants. For instance, plants absorb nutritional potassium through low-affinity, inward-rectifying potassium channels, such as *AtAKT1* genes in *Arabidopsis*. Cesium inhibits voltage-dependent, inward-rectifying potassium channels, and subsequently induces potassium starvation in plants. When potassium in the soil is depleted, plants increase the expression of voltage-independent Ca^2+^ channels (VICCs), such as *AtKUP* genes (e.g. *AtKUP3* and *AtHAK5*) in *Arabidopsis*. VICCs are mainly responsible for cesium uptake by plants, by accumulating cesium under potassium deficient conditions [4, 6–10]. In addition, a recent study showed that Zinc-Induced Facilitator-Like 2 (ZIFL2), a member of the Major Facilitator Superfamily (MFS) of membrane transporters, uptakes K^+^ and Cs^+^ in *Arabidopsis thaliana* [11]. Furthermore, Adams et al. (2013) reported that cesium inhibits the growth of plants via the jasmonate (JA; a lipid-based hormone) signal pathway, which regulates a wide range of processes in plants [12]. Dräxl et al. (2013) showed how internal accumulation of cesium can be suppressed in plants via a mutation in a type of SNARE (Sec22p/SEC22) protein that plays a primary role in vesicle fusion [13]. By applying suppression-subtractive hybridization (SSH) and RT—PCR methods, some Cs-stress responsive genes were identified and analyzed [14]. However, with regards to the response to cesium toxicity, a general overview of gene expression profiles, regulatory layers of gene expressions, and the identification of cesium-specific genes has not been fully established.

MiRNAs are a class of small non-coding RNA molecules, 21–22 nucleotides in length, which function in the post-transcriptional regulation of gene expression [15]. Primary transcripts of miRNA genes (pri-miRNAs) are processed into precursor-miRNA (pre-miRNAs), and further matured into 21–22 nt miRNAs by a highly coordinated protein complex, known as a microprocessor. In plants, miRNAs play fundamental roles in the regulation of stress responses, development, differentiation, embryogenesis, reproduction, and signaling [16]. To date, over 4000 miRNAs have been identified in plants, and some of their specific targets and functions are highly correlated to environmental stresses [16, 17]. Notably, some of miRNAs are up-and-down regulated by the concentration changes of essential trace elements in soil (nutritional stresses). For instance, in *Arabidopsis thaliana* and *Brassica napus*, miR395 is over-expressed under sulfate-starvation conditions [18, 19]. In response to nitrogen concentration, miR167 and miR393 involve in the regulation of root development and growth [20, 21]. The expression of *MiR398* is dramatically decreased in response to excessive Cu, resulting in accumulation of *CSD1* and *CSD2* genes for Cu/Zn superoxide dismutases [22, 23]. A group of highly conserved miRNAs includes miR159, miR169, miR172, miR173, and miR394 are differentially expressed under Fe-deficiency and many miRNAs harbor IDE1/IDE2 motifs, Fe-deficiency responsive cis-acting elements, in their promoters [24]. Likewise, non-essential (non-nutritional) trace elements such as heavy metals in soil influence the expression of miRNAs in plants. Several recent studies have described the active involvement of miRNAs that counteract heavy metal toxicity by regulating various transcription factors and protein coding genes [19, 25–27]. However, knowledge of the detailed roles of miRNAs in regulating the factors and proteins for heavy metal resistance is still rudimentary. Here, we show that the whole expression profile of miRNAs can be affected by high concentrations of cesium. The high concentration of cesium dramatically increased both the levels of pri-miRNAs and target mRNAs, indicating a negative effect of cesium on both miRNA processing pathways, and the mRNA clearance pathway.

## Materials and Methods

### Plant materials and growth conditions

Col-0 wild type seeds were grown on Murashige and Skoog (MS) medium (1% sucrose and 0.8% agarose) after sodium hypochlorite (2%) sterilization. The seeds were stratified at 4°C for 1 day in dark and transferred to a growth chamber (18-h light and 6-h dark at 22°C). Seven or 14 day-old seedlings were transferred to MS medium as a control, or MS medium containing 10 mM KCl or 10 mM CsCl, followed by 3 days of incubation.

### RNA extraction and qRT-PCR

Total RNAs were extracted from *Arabidopsis* seedlings using RNeasy Plant Mini kits (Qiagen), followed by reverse transcription using the iScript cDNA Synthesis Kit (Biorad). qRT—PCR was carried out with the synthesized cDNAs, using the CFX384 Touch Real-Time PCR Detection System (Biorad). Relative quantities of transcripts were obtained by calibrating the target genes’ threshold cycles (Ct) with Ct value of ACTIN, using the calculating formula 2^(-ΔΔCt)^. The experiments were performed with three independent biological samples. Primers for qRT-PCR are described in S1 Table.

### Small RNA extraction and northern blot analysis

Total RNAs were isolated from *Arabidopsis* seedlings using the reagent Trizol (Invitrogen). The extracted aqueous phase was precipitated twice with isopropanol (100 and then 75%) and resolved in 50% (w/v) formamide after treating with DNase. Purified RNAs were resolved on 12.5–15% denaturing polyacrylamide gel (National Diagnostics), then transferred to a nylon membrane (Amersham). The 5′ end-labelled DNA probes were applied for 12 h to achieve hybridization of blots (Ambion). Blots were washed twice with SSC (2×)/SDS (0.1%) for 20min each. Hybridization signals were detected with Typhoon trio phosphoimager (GE Healthcare).

### Deep sequencing and analysis of small RNAs

Small RNA library construction and small RNA analysis with a Col-0 control, and 10 mM KCl or 10 mM CsCl treated seedlings was performed by Macrogen Ltd., Korea. The miRNA expression levels (TPTM) in the indicated samples were calculated by normalizing miRNA counts with the total number of clean reads in the small RNA libraries.

### Immunoblot analyses

KCl or CsCl treated seedlings with negative controls were disrupted in liquid nitrogen with a pestle and mortar, and mixed with 5× SDS sample buffer for 10min. After boiling at 100°C, the extracts were centrifuged at 13,000 rpm for 10 min. The supernatants were resolved on 12% SDS—PAGE, and the resolved gels were transferred to a PVDF membrane (Biorad). Blots were detected with α-HYL1 antibody (dilution 1:5000, Cho et al., 2014), α-SERRATE antibody (dilution 1:5000, Cho et al, 2014), α-AGO1 antibody (dilution 1:5000, Agrisera), and α-ACTIN antibody (dilution 1:5000, Agrisera), respectively.

## Results

### Phenotypes of *Arabidopsis thaliana* under excessive potassium or cesium stress

**Fig 1:**
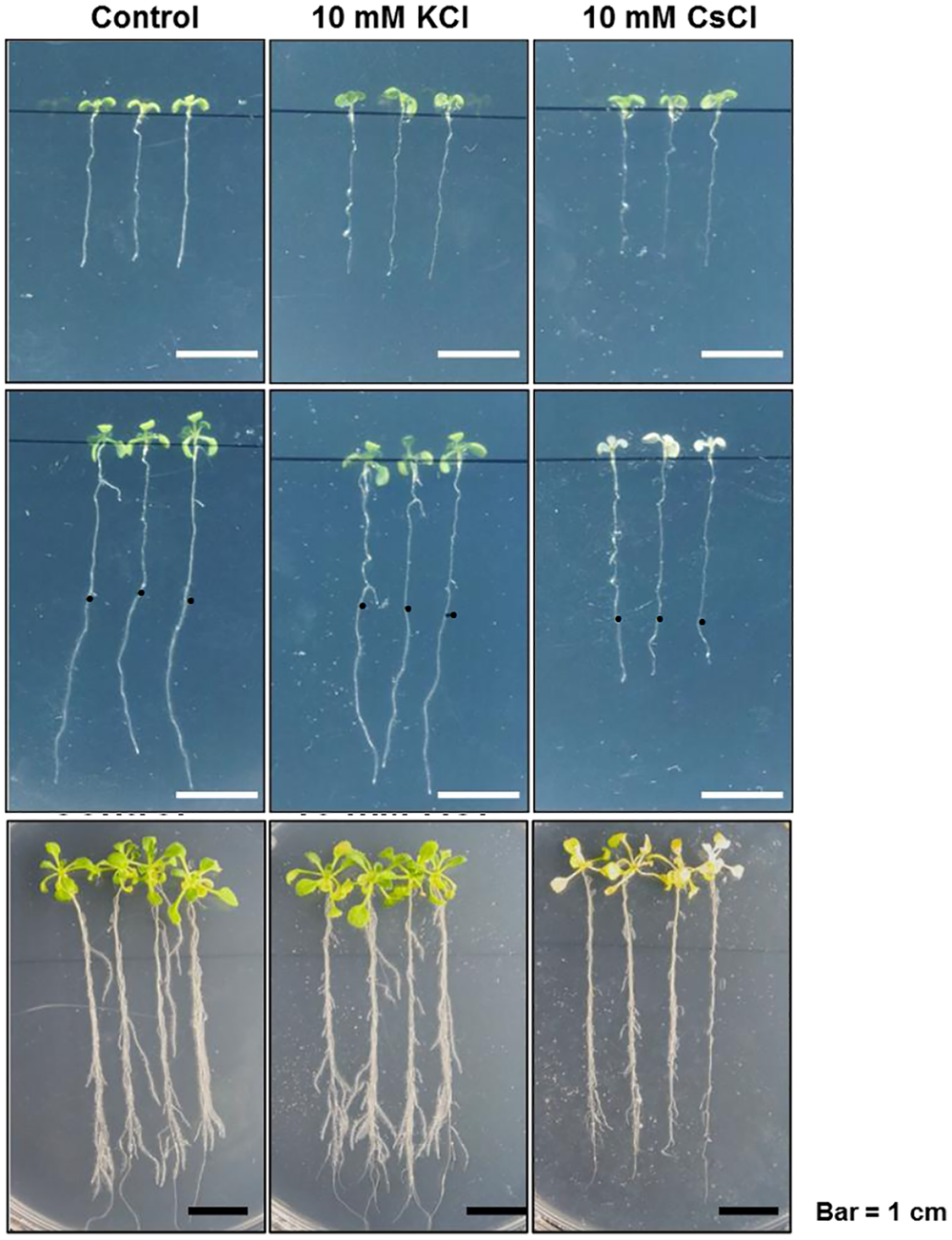
Phenotype of seedlings grown for 3 days on MS medium, amended with/without KCl (10 mM) or CsCl (10 mM). Upper panel: plantlets transplanted from MS medium. Middle panel: 3 days after the plantlets were transplanted to MS medium, with the amended chemicals. Black dots indicate the end point of roots in the upper panel. Lower panel: Two-week-old seedlings were transplanted to KCl or CsCl supplemented MS medium for 3 days.

The detrimental effect of high cesium concentrations on the growth and development of *Arabidopsis thaliana* has been previously reported. Seed germination rate is dramatically reduced at a concentration of 1 mM cesium, and plantlets are in general unable to survive after a few days of this treatment. Treatment with a lower concentration of cesium, between 500 to 700 μM, causes seedlings to start showing signs of chlorosis and retarded root growth after three weeks [14]. To monitor these effects, seedlings were grown on MS medium for one week, before transferring to a cesium-excessive medium (10 mM), or a potassium-excessive medium (10 mM) for three days. Under the cesium treatment condition, the root development of Col-0 plants (wild-type) was severely arrested, than that of plants grown on MS medium and MS medium with 10 mM KCl (Fig 1).

Not only was the elongation of the primary root and root hairs retarded, but chlorosis—the condition where leaves have insufficient chlorophyll—was more extensive in the seedlings grown in 10 mM CsCl (Fig 1 middle panel). Compared to young seedlings, under the same condition the progression of chlorosis in two weeks-old seedlings was confined to a particular part of the leaves, implying that younger plantlets are more susceptible to cesium-toxicity (Fig 1, bottom panel). This result clearly demonstrates the pernicious effects of excessive cesium on the development of plants.

### Length distribution and population of small RNAs in seedlings under KCl or CsCl stress

**Fig 2:**
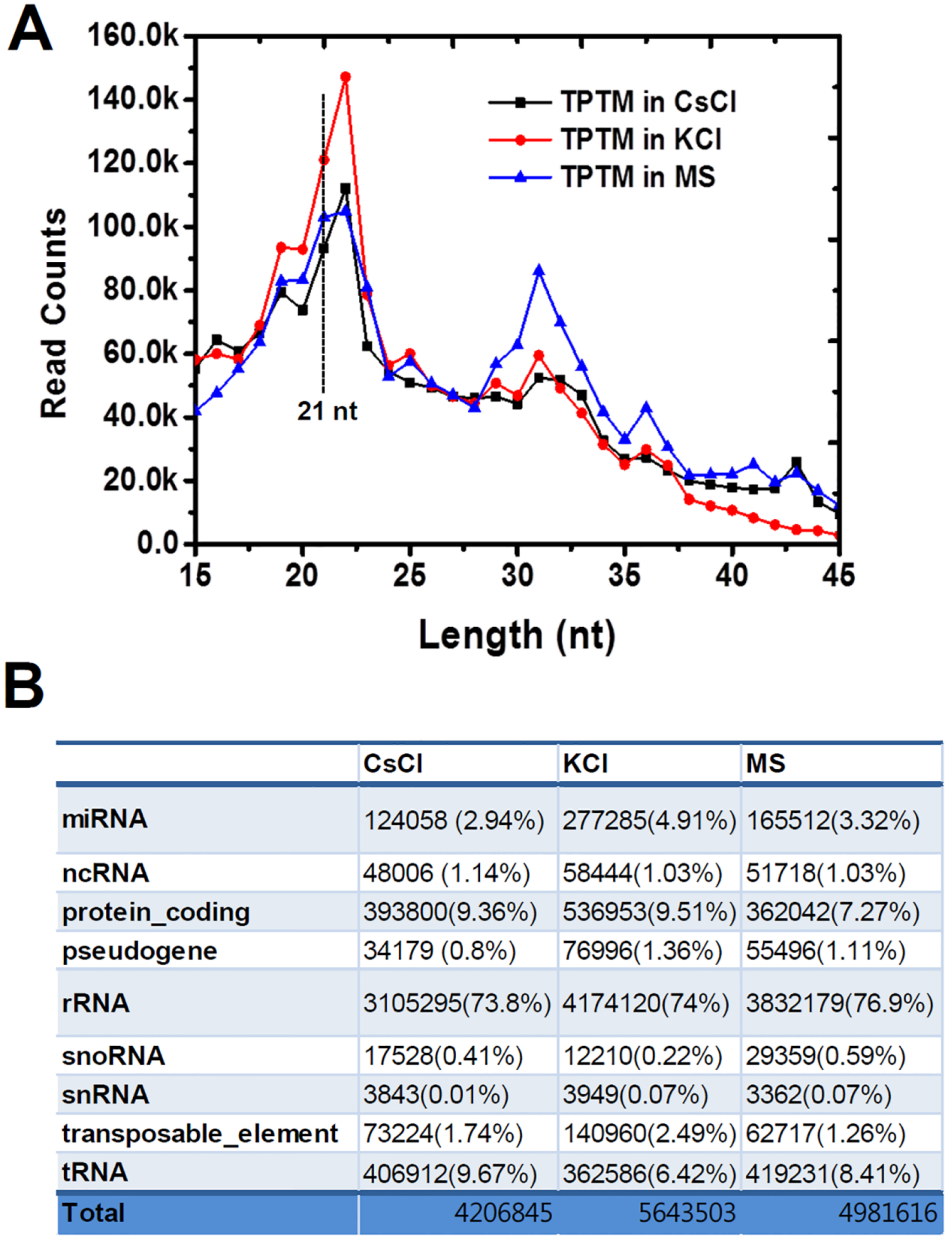
Size distribution and population of small RNAs from control seedlings, CsCl-treated seedlings, and KCl-treated seedlings. A. Size distribution of miRNAs. B. Summary of small RNA profiles in the tested seedlings.

To retain plant viability, two week old seedlings were transiently treated with potassium (10 mM) or cesium (10 mM). Total small RNAs were purified and sequenced using the Illumina platform, we generated 73.2. 78.9, and 94.4 million clean reads that perfectly matched the Arabidopsis genome from the small RNA populations in control seedlings, CsCl-treated seedlings and KCl-treated seedlings. With this sequence information, we analyzed the size distribution of small RNAs, including miRNAs. Small RNAs of over 15 nucleotides and below 45 nucleotides (minus the adaptor sequences and barcode), were acquired for further analysis. Most of the small RNA fragments were within the range of 15 nt to 24 nt, which accounts for 93% of the acquired clean reads. The total sequence reads of the small RNAs from each sample were adjusted by transcripts per 10 million (TPTM), and plotted on a linear graph (Fig 2A).

Under our experimental conditions, small RNAs of 21 nt and 22 nt were highly abundant in both the control seedlings and the stress-treated seedlings. The 21 nt and 22 nt fragments accumulated significantly in KCl-treated seedlings, compared to the control and CsCl-treated seedlings. We also observed a second major peak in control seedlings, at 31 nt. The TPTM of the 31 nt is significantly lower in both CsCl- and KCl-treated seedlings than in the control. Interestingly, this pattern of small RNA distribution—two peaks at 22 nt and 31 nt—was not monitored in our previous deep sequencing analysis of the control seedlings, in which small RNA of 21-nt and 24-nt sizes are predominant (Cho et al., 2014). We assume that this might be caused due to the difference in the growth conditions and sampling time. The small RNAs were classified into miRNA, non-coding RNA, rRNA, snRNA, snoRNA, tRNA, and other RNA according to their origins, and the proportion of the total that fell into each class was calculated (Fig 2B). A slight increase in small RNAs (1.3%) derived from tRNA was observed under the CsCl-treated condition, while small RNA decreased (2%) under the KCl treatment. The primary group of small RNA, that which was shorter than 45-nt, mostly originated from rRNA. This was also slightly reduced in both the CsCl-treated and KCl-treated seedlings. In general, neither the length distribution of small RNA—except the unusual accumulation of 32 nt RNA in the control seedlings—nor the origin proportion was notably changed under stress conditions. In fact, the lengths of most miRNAs are in the range of 21 nt and 22 nt. Hindrances to miRNA processing—for instance by a mutation in the miRNA microprocessing components—easily results in a dissipation of the fragments in a small RNA population [28]. However, in this study, the 21 nt and 22 nt fragments were not affected by the CsCl treatment, implying that the processing accuracy of miRNAs may not be significantly influenced by Cs-toxicity. Next, we further investigated the expression patterns of small RNA sequences from miRNA genes.

### Expression profiling of defined miRNAs responding to CsCl or KCl treatment

**Fig 3:**
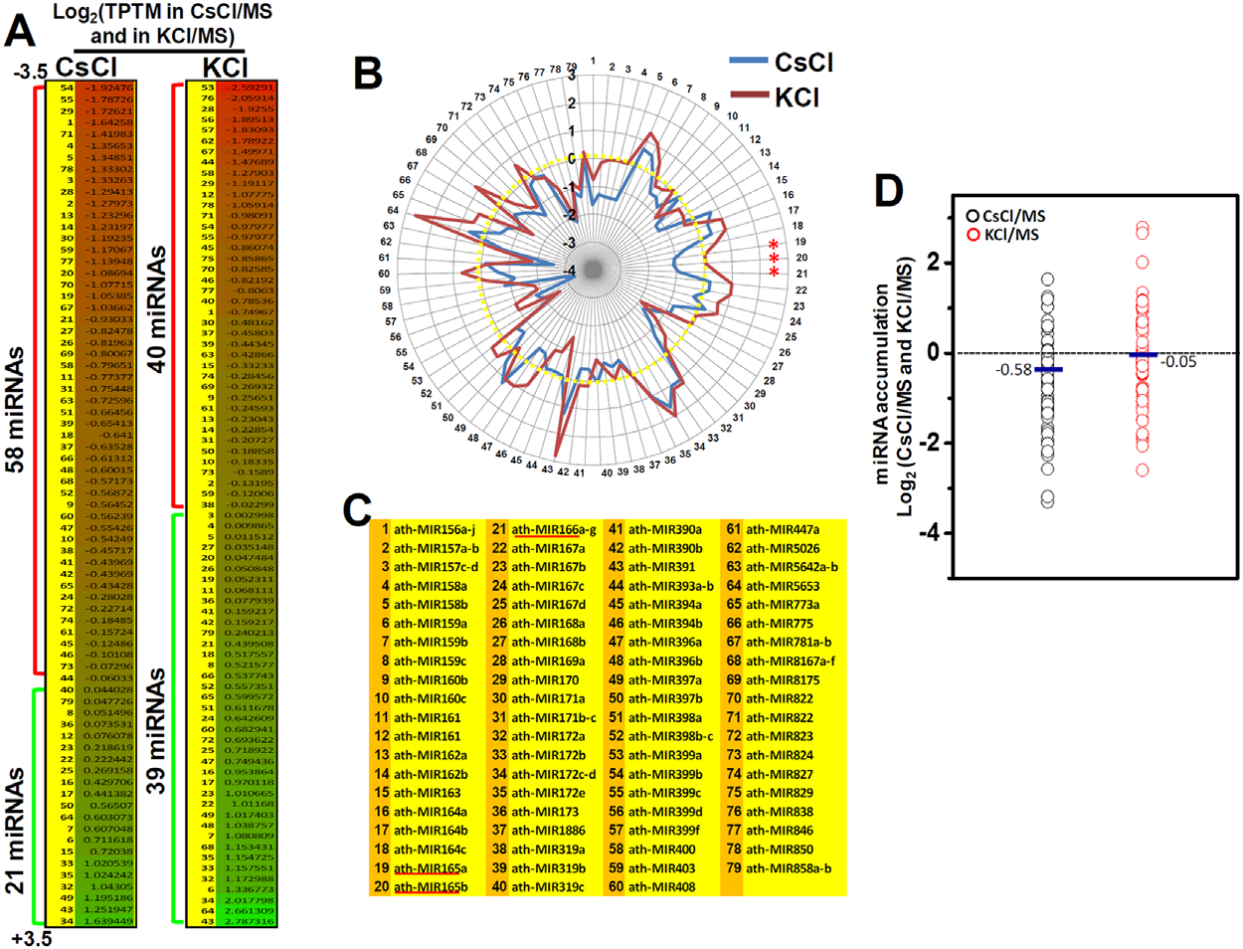
The profiles of miRNAs in CsCl-treated and KCl-treated seedlings. A. Log_2_ (TPTM in salts/MS) heat map of trimmed miRNAs from CsCl-treated and KCl-treated seedlings. B. Radial chart of log_2_ (TPTM in salts/MS) trimmed miRNAs from CsCl-treated and KCl-treated seedlings. Red line represents KCl-treated seedlings. Blue line represents CsCl-treated seedlings. C. List of miRNAs. Each number matches the number in the heat map (A) and the radial chart (B). D. The average log_2_ (TPTM) values of the miRNAs of CsCl-treated or KCl-treated seedlings versus control seedlings.

The small RNA sequences from the CsCl-treated and KCl-treated seedlings were analyzed for the presence of previously characterized *Arabidopsis* miRNAs. A total of 434 miRNAs from the combined RNA sequence reads were identified, but 350 of these were lower than 5 TPTM, and thus excluded. Amongst the 79 miRNAs that had total expression of at least 5 TPTM, 58 (73%) and 40 (51%) of the miRNAs showed reduced expression in CsCl-treated seedlings and KCl-treated seedlings, respectively. Most of the miRNAs, whose expressions declined in CsCl-treated seedlings (65%) and KCl-treated seedlings (38%), were reduced by more than 50% (log_2_ ≥ 0.25) compared to the wild type. On the contrary, the levels of 21 miRNAs (27%) and 39 miRNAs (49%) were increased in CsCl- and KCl-treated seedlings, respectively. Compared to the control seedlings (17540 reads), the read number of 79 miRNAs was reduced by around 30% in CsCl-treated seedlings (12809 reads), whereas the number was increased by around 37% in KCl-treated seedlings (24096 reads) (Fig 3A, S1 Fig).

The average Log_2_ (TPTM in salt/MS) values of the miRNAs were -0.58 for CsCl-treated seedlings and -0.052 for KCl-treated seedlings (Fig 3D). These results clearly show the detrimental effects of CsCl on the expressions of miRNAs.

From the sequencing information, we found that CsCl or KCl treatment significantly affected several well-defined miRNA families. While the expressions of 14 families (miR156/miR157, miR158, miR160, miR162, miR165/miR166, miR168, miR169, miR171, miR390, miR393, miR394, miR396, miR398, and miR399) were dramatically reduced, 3 families (miR159, miR167, and miR172) were up-regulated in CsCl-treated seedlings. In the case of KCl treatment, the miRNA counts of 4 families (miR156/miR157, miR169, miR394, and miR399) were reduced, whereas 9 families (miR159, miR164, miR165/miR166. miR167, miR168, miR172, miR396, and miR398) were notably increased (Fig 3B, S2 Fig).

Several miRNA families were significantly lower in both CsCl-treated and KCl-treated seedlings (miR156, miR169, miR170/miR171, and miR399). Specifically, the miR156 family consists of 10 genes (miR156a—miR156j) and targets 10 members of the *SQUAMOSA PROMOTER BINDING PROTEIN LIKE* (*SPL*) transcription factors [29, 30], thereby controlling developmental timing. The expression of these miR156 genes was measured at a total 2536, 871, and 1918 reads per TPTM in the control, CsCl-treated and KCl-treated seedlings, respectively (Fig 3B, number 1). The miR169 family contains 14 genes (miR169a - miR169n) and mainly functions in regulating plant responses against salt and drought stresses [31–33]. Although they were expressed at very different frequencies, the overall reduction in the expression of miR169 genes was observed in both CsCl-treated (3-fold) and KCl-treated seedlings (3.5-fold) (Fig 3B, number 29). Likewise, the miR399 family consists of 15 genes (miR399a - miR399o) and mostly contributes to the maintenance of phosphate homeostasis [34, 35]. In our analysis, we detected the expression levels of only 5 genes (miR399a, b, c, d, and f) which were significantly suppressed in both CsCl-treated (5-fold) and KCl-treated seedlings (3.2-fold) (Fig 3B, number 57–61). On the other hand, the expressions of two miRNA gene families, miR159 and miR172, were dramatically increased in stress-treated seedlings. The miR159 family contains 3 genes, the expression of which completely silences two *GAMYB-like* genes in the vegetative tissues. Compared to the control seedlings (1131 reads), miR159a accumulated significantly in both CsCl-treated seedlings (1852 reads, 163%) and KCl-treated seedlings (2857 reads, 252%) (Fig 3B, number 6–8). In the case of miR156b, the read counts were 1242, 895, and 587 in KCl-treated seedlings (211%), CsCl-treated seedlings (152%), and in control seedlings (587 reads), respectively. The five genes belonging to the miR172 family—which mainly represses AP2 transcription factor genes that modulate flowering time and floral development—were also increased by about two fold in both of the stress treatments (Fig 3B, number 33–36).

**Fig 4:**
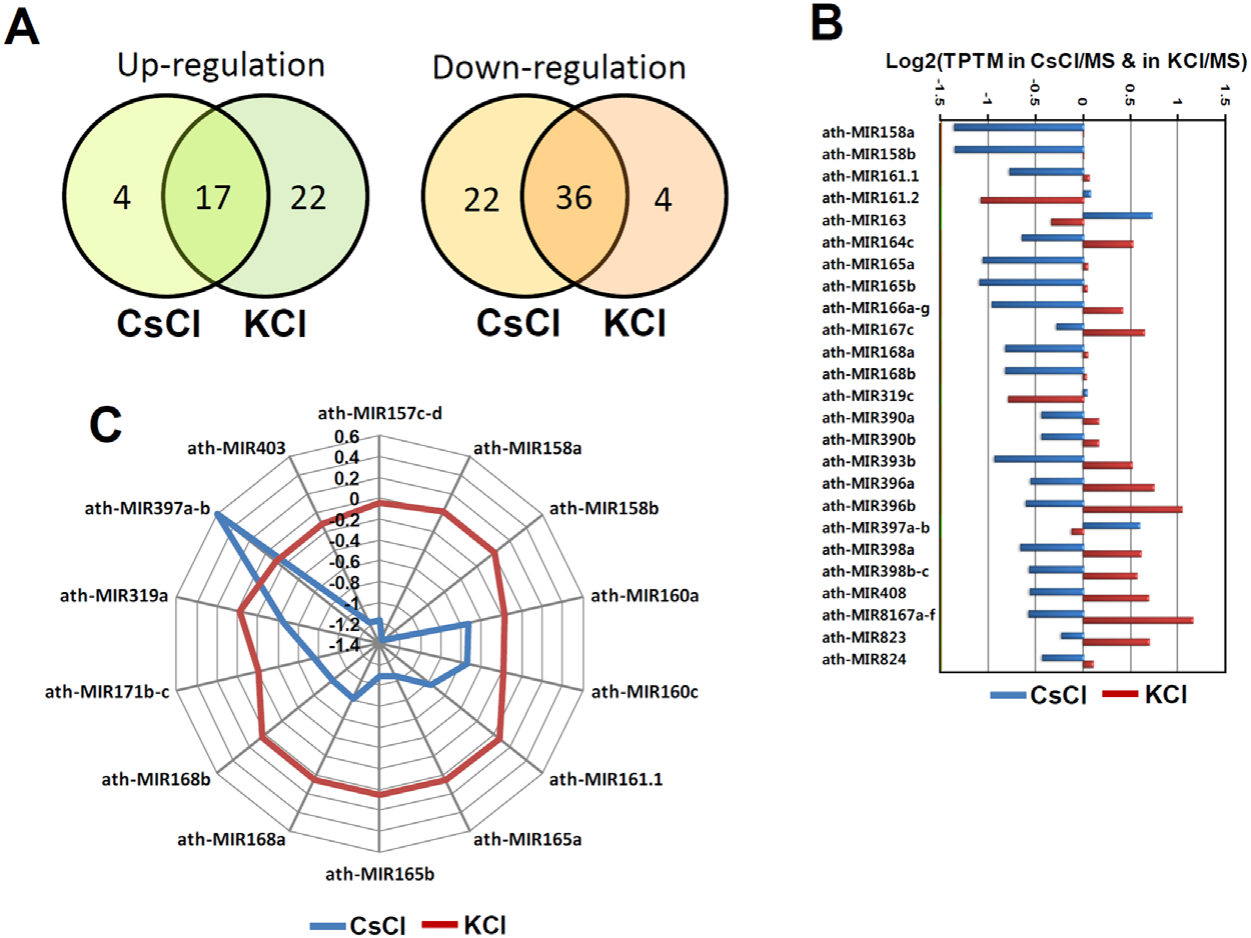
Differentially regulated miRNAs under Cs-toxicity. A. Comparison of up-or-down regulated miRNAs from CsCl-treated and KCl-treated seedlings. B. Differentially expressed miRNAs, which are inversely correlated between CsCl-treated and KCl-treated seedlings. C. Radial chart of miRNAs which are specifically altered by CsCl treatment.

The profiles of the down-regulated miRNAs showed a 46% overlap between the CsCl-treated seedlings and KCl-treated seedlings (Fig 4A).

Besides, the profiles of up-regulated miRNAs overlapped less than 21% between the two treatment conditions (Fig 4A). In order to summarize the specific effect of cesium on the expression of miRNAs, we investigated the response of differentially expressed miRNAs from the CsCl- and KCl-treatments. As shown in Fig 4B, 18 miRNA families (21%) were differentially expressed in the CsCl-treated seedlings, and more interestingly, the expression of the 18 miRNAs were inversely regulated in KCl-treated seedlings. Fifteen of them were specifically reduced in CsCl-treated seedlings, but increased in KCl-treated seedlings. In contrast, only 4 of them were reduced in KCl-treated seedlings, but were more highly expressed in CsCl-treated seedlings (Fig 4B). The 18 cesium-responsive miRNAs include many conserved and functionally well-defined miRNAs. Next, we further analyzed the expression profiles of the miRNAs and found 10 miRNA families that barely responded to KCl treatment. This indicated the presence of Cs-specific miRNAs. As shown in the radial chart in Fig 4C, expression of the miR157, miR160, miR165, miR168, miR171, miR319, and miR403 families was decreased by around 80% to 140% in CsCl-treated seedlings. However, the expression of these same families was not altered in KCl-treated seedlings. In contrast, expression of the miR397 family was about 1.2-fold increased by CsCl treatment. Taken together, these results clearly suggest that plants differentially modulate at least 18 miRNAs in response to Cs-toxicity, ten of which are irrelevant to KCl stress resistance.

### Expression levels of a subset of pri-miRNAs are increased by CsCl treatment

**Fig 5:**
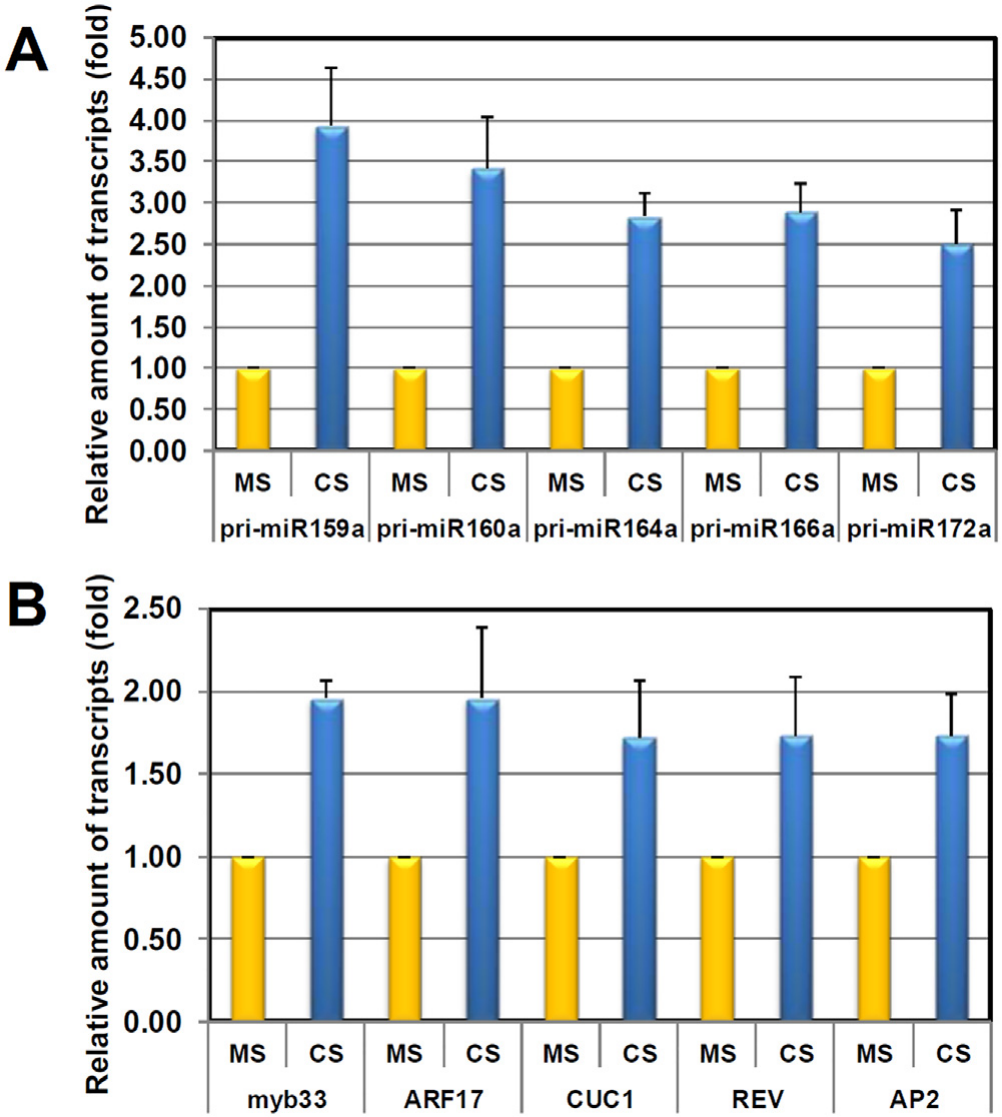
qRT-PCR expression analyses of selected pri-miRNAs (A) and miRNA target transcripts (B) in control or CsCl-treated seedlings. Relative amounts of pri-miRNAs or mRNA levels were obtained by dividing the expression level of the mutant by the control value. Three biological samples were used.

The results of deep sequencing (Figs 3 and 4) showed the adverse effect of Cs-toxicity on miRNA biogenesis. Previously, many studies reported on the inverse correlation between the levels of pri-miRNAs and mature miRNAs. Defects in the miRNA processing pathway lead to dramatic accumulations of pri-miRNAs and subsequent reductions in the levels of mature miRNAs [36–38]. On the contrary, when miRNA processing is accelerated, levels of pri-miRNAs drop, and mature miRNAs accumulate [39]. Transcripts of many miRNA genes (*MIR*) are spatially and temporally modulated by diverse environmental and cellular signaling factors, such as hormones and both biotic and abiotic stresses [40–43]. To investigate which regulatory layers—the miRNA processing pathway or *MIR* transcriptions—were disrupted by Cs-toxicity, we determined pri-miRNA transcript levels by qRT-PCR (Fig 5A). Intriguingly, transcripts of all tested pri-miRNAs accumulated significantly more in Cs-treated seedlings than in control seedlings (Fig 5A).

At first glance, in accordance with the reduced levels of miR160 and miR166 (Fig 3B), the accumulations of pri-miR160a (~ 3.5-fold) and pri-miR166a (~ 3-fold) seemingly reflected the interference with the miRNA processing pathway. However, the dramatic accumulations of pri-miR159a (~ 4.5-fold), pri-miR164a (~3-fold), and pri-miR172a (~2.5-fold) were proportional to the levels of mature miR159 (~ 2-fold), miR164 (~ 1.6-fold) and miR172 (~ 2-fold) present. Moreover, the increased ratios were approximately correlated, implying that the effect of Cs-toxicity is complex (Fig 5A). Although these results—regarding which of regulatory layers, the miRNA processing pathway or MIR transcription, is disrupted—are difficult to analyze, it is apparent that the abnormal accumulation of pri-miRNAs is caused by Cs-toxicity. The reason for the simultaneous accumulation of precursors and mature miRNAs (miR159 and miR172) remains unclear. We carefully speculate that might be caused by 1) uncoupling of *MIR* gene expression and processing efficiency or 2) differential degradation of miRNAs. First, similar to Fe-induced *MIRNA* genes, if some of *MIRNA* genes (miR159 and miR172) are dramatically up-regulated by a high concentration of Cs, then the final products, mature miRNAs, can be subsequently increased or maintained, irrespectively of processing retardation. On the contrary, in the cases of *MIRNA* genes which are uninduced by Cs-toxicity, then the retardation in processing pathway directly leads to a clear reduction of mature miRNAs. Second, it can be considered that the differential degradation of miRNAs by specific RNAs such as SDN1 [44, 45]. In order to clarify the speculations, we have to further investigate by promoter analysis and/or miRNA half-life assay under Cs-toxicity.

### Expression levels of a subset of target mRNAs are increased by CsCl treatment

MiRNAs mainly direct RISC complex to clip off target mRNAs, and thus the levels of target mRNAs are also inversely proportional to the levels of their complementary miRNAs [46]. For instance, we speculated that the increased levels of miR159, miR164, and miR172 should lead to a decrease in the target mRNAs, *MYB3*3, *CUC1*, and *AP2*, respectively. Conversely, the accumulated levels of *ARF17* and *REV* should be monitored due to the reduction of miR160 and miR166. To confirm this inverse correlation under Cs-toxicity, we determined target mRNA transcript levels by qRT-PCR (Fig 5B). Surprisingly, transcripts of all tested mRNAs—*MYB33*, *ARF17*, *CUC1*, and *AP2*—accumulated more in Cs-treated seedlings than in control seedlings (Fig 5B). All tested target mRNAs were increased en bloc by about 1.5-fold to 2-fold, irrespective of the various levels of guiding miRNAs present. Under normal circumstances, and in response to a variety of stimuli, plants struggle to retain the homeostatic balance between miRNAs and target mRNAs appropriate to their development and growth [34, 47]. However, this balance seems to collapse under the stress of Cs-toxicity, which overwhelms the plants’ adaptive plasticity.

### Cs-toxicity disrupts both the miRNA processing pathway and AGO1 expression

**Fig 6:**
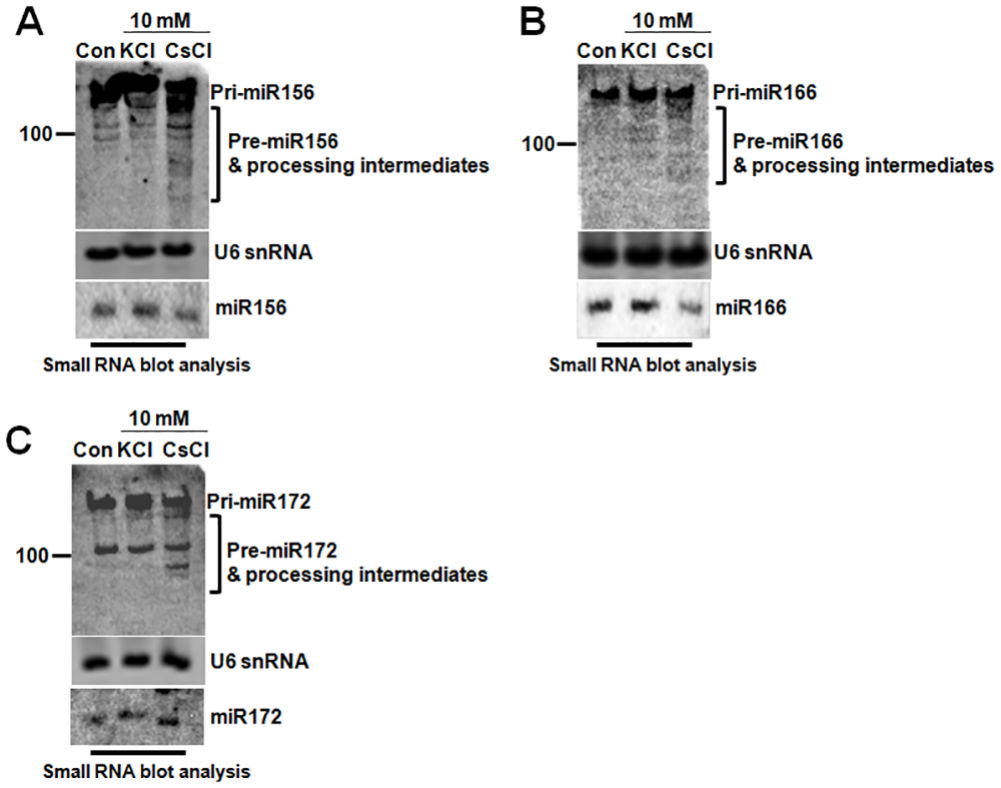
Cs-toxicity hinders the processing of pre-miRNAs. A. The processing pattern of pri-miRNA156. B. The processing pattern of pri-miR166. C. The processing pattern of pri-miR172. Small RNA blot analyses display the highly accumulated processing intermediates in CsCl-treated seedlings. U6snRNA was used as the loading control.

Given these results (Fig 5), it is reasonable to speculate that Cs-toxicity might hinder both RISC complex-mediated mRNA clearance and the miRNA processing pathway. To test this hypothesis, we monitored the integrity of the miRNA processing pathway using small RNA blot analysis. As shown in Fig 6A, pri-miR156 and pre-miR156 fragments can be observed in an RNA blot analysis. In the blot, the intermediate fragments below 100 nt were more increased in CsCl-treated seedlings than in the control and KCl-treated seedlings.

Likewise, the intermediate fragments of pri-miR166 and pri-miR172 were clearly detected in CsCl-treated seedlings (Fig 6B and 6C). In two microprocessor mutants, *hyl1-2* and *dcl1-9*, pri-miRNAs were highly accumulated, while accumulation of both the intermediates (pre-miRNAs, remnants) and mature miRNAs were decreased dramatically. This is because these two proteins are essential to the first processing step [36]. Under Cs-treatment, the pattern of the accumulated intermediate fragments was slightly different from the inverse correlation between pri-miRNAs and the processing products. Although the processing interference of pri-miR172 conflicted with the levels of miR172—if the processing is interrupted, mature miR172 shouldn’t accumulate further—these results obviously show that Cs-toxicity retards the processing of pri-miRNAs into mature miRNAs. We carefully speculated that the conflict might occur for an unstudied reason: the increase in the expression of *MIR172* due to Cs-treatment.

**Fig 7:**
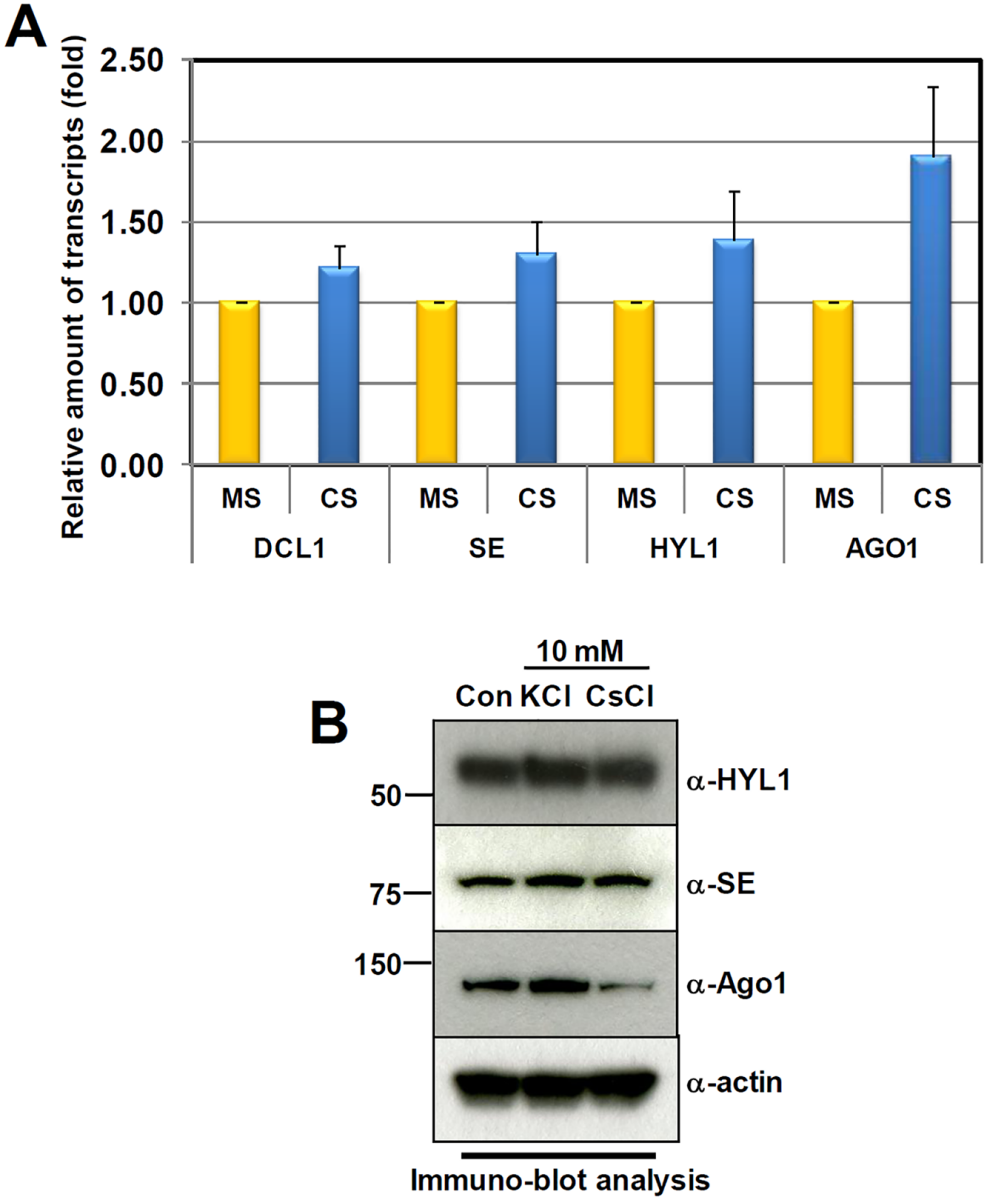
Cs-toxicity enhances the proteolytic degradation of AGO1. A. qRT-PCR analysis of *HYL1*, *SE*, *DCL1*, and *AGO1*. B. Immuno-blot analysis shows the level of HYL1, SE, DCL1, and AGO1 under the treatments of indicated chemicals. The α-actin antibody was used as the loading control.

Next, to understand how target mRNAs are accumulated in Cs-treated seedlings, regardless of the levels of complementary miRNAs, we investigated the expression of a major RISC complex gene, *AGONOUTE 1* (*AGO1*), using qRT-PCR (Fig 7A).

We found that in comparison to the control seedlings, *AGO1* transcripts are increased around 2-fold by CsCl treatment. Recently, it has been reported that AGO1 is an unstable protein that is degraded by autophagy during virus infection [48]. We therefore performed an immune-blot analysis to test the integrity of AGO1 under Cs-toxicity. Interestingly, compared to the stable HYL1 and SE (two important RNA-binding proteins in miRNA processing), AGO1 was significantly reduced in CsCl-treated seedling (Fig 7B). Considering the amassed *AGO1* transcripts, AGO1 seemed to be destabilized through a proteolytic pathway, possibly autophagy. In conclusion, our results show that Cs-toxicity may retard the processing of pri-miRNAs into miRNAs, and enhance the proteolytic destabilization of AGO1. This subsequently damages plants, both in terms of miRNA homeostasis and flexible regulation of mRNAs.

## Discussion

Cesium is indisputably toxic to the development and growth of plants. The detailed mechanisms of Cs-toxicity have been reported to be related to potassium metabolism, including potassium uptake, potassium starvation, and potassium-dependent enzyme activities [2, 4, 7]. Many subsequent studies have described, in detail and from a variety of perspectives, the biological responses of plants to Cs-toxicity [49–52]. However, there is currently neither a profile of whole transcripts nor of miRNAs has been established for Cs-toxicity in plants. Because of the importance of miRNAs in plant plasticity responses to environmental stimuli, many recent studies have described the close relation between the surplus or starvation of mineral elements such as N, P, S, and Cu and differential expressions of miRNAs [53–56]. For instance, a group of miRNAs were suppressed during N-starvation, but rather increased during P, S, Cu-starvation, suggesting that this group of miRNAs might be involved in signal transduction under nutrient-deprived conditions [54–56]. Furthermore, several reports have described the effects of heavy metals (HMs) such as Mn, Cd, Hg, Al, Ti, and As on miRNA biogenesis in plants [27, 57–61]. In response to those HMs, plants actively increase and decrease expression of miRNAs, so that various transcription factors and protein coding genes can be flexibly modulated to aid survival. However, knowledge of the role of miRNAs in controlling genes for HM metabolism is still rudimentary.

Based on informatics, and biochemical and molecular analyses, we here have demonstrated the adverse effects of Cs-toxicity on miRNA-mediated gene silencing. We found that of the 79 verified miRNAs, 58 were down-regulated, while 21 were up-regulated during Cs-toxicity. Among the down-regulated miRNAs, it is worthwhile to take note of the miR165/166 family—consisting of seven individual *MIR166* and two *MIR165* genes—which specifically responded to CsCl-treatment. Considering the wide range of downstream target genes (e.g. HD-ZIP III proteins), for the development of shoots and roots, the reduction of miR165/166 and subsequent accumulation of the target genes might account for the developmental and growth defects under Cs-toxicity (Fig 1). Not only does this indicate the presence of a disturbance in miRNA biogenesis under Cs-toxicity, but also suggests the existence of two possible molecular mechanisms underlying the disturbance. First, Cs-toxicity seems to hinder the processing speed of pri-miRNAs into mature miRNAs. Considering the stability of HYL1 and SERRATE, Cs-treatment is the likely cause of the interference with pre-miRNA processing, irrespective of the microprocessor deficiency seen in *hyl1-2*, *se-1*, and *dcl1-9* (Fig 7B). We tentatively speculate that Cs-toxicity may somehow delay the catalytic activity of the microprocessor. Thus, the variations in the levels of different miRNAs could be attributed to order-of-magnitude differences in processing retardation and differential *MIR* gene expressions. Second, Cs-toxicity impairs RISC complex-mediated gene silencing, by destabilizing AGO1. As previously mentioned, AGO1 is the major component of the RISC complex, by which mRNAs can be cleaved under the guidance of complementary miRNAs. Therefore, the significant reduction of AGO1 in Cs-treated seedling indicates that many target mRNAs may flee from the decaying RNA-mediated mRNA system, possibly resulting in developmental derangements. Although we cannot clearly rule out the possible disruption of the other mRNA decaying pathways by Cs-toxicity such as deadenylation-mediated mRNA decaying, non-sense mediated mRNA decaying and ARE-mediated mRNA decaying, at the moment we prefer to explain the accumulation of target mRNAs in the context of AGO1 reduction and processing retardation. In conclusion, in spite of the physical and chemical mimicry of K by Cs, we suggest that Cs has a distinctive and noxious effect on the RNA-mediated gene silencing pathway; miRNA processing, and AGO1-dependent mRNA clearance.

In addition to the toxicity of Cs metal itself, radioactive Cs-137, a major contaminant from the Chernobyl and Fukushima disasters, is still persistently spreading across the globe, threatening global health and environmental systems. It has been found in many agricultural products, enhancing the uptake of radioactive cesium into the food chain, and increasing concerns over global food safety. To manage this radiological contamination, a variety of agricultural countermeasures, including phytoremediation and Cs-free crops, have been discussed as high priority issues. Hence, our study of this novel perspective of Cs-toxicity may provide a clue for the development of biological countermeasures. Furthermore, for the sake of both human and cattle health, the understanding of Cs-toxicity’s effect on the RISC complex may contribute to our understanding of the multiple ways in which Cs intoxication may affect larger organisms consuming contaminated food or fodder. In the future, we plan to enrich our understanding of the causes of Cs-toxicity, by investigating how Cs hinders the RNA-mediated gene silencing pathway.

## Supporting Information

S1 Dataset (XLSX)

S1 Fig Total read counts of various sized small RNAs in the control seedlings, CsCl-treated seedlings, and KCl-treated seedlings. (DOCX)

S2 Fig Log_2_ (TPTM in salts/MS) heat map of trimmed miRNAs from CsCl-treated and KCl-treated seedlings. (DOCX)

S1 Table Primers for qRT-PCR. (DOCX)
