# Supplementary material for: Cesium Toxicity Alters MicroRNA Processing and AGO1 Expressions in Arabidopsis thaliana
Source: PLoS One. 2015 May 6;10(5):e0125514. doi: 10.1371/journal.pone.0125514 (PMC4422737; doi:10.1371/journal.pone.0125514)
Supplement: S1 Table — (DOCX) [file pone.0125514.s004.docx]

Supplementary Table 1

**qRT-PCR**

**Pri-miR159a**

5′-AGCAGGGTAAAGAAAAGCTGC-3′/5′-AGGGCAAGTTAAAGCTCCTGA-3′

**Pri-miR172a**

5′-GTCGTTGTTGGCTGCTGTG-3′/5′-TCGTTGATTGCCGATGCAG-3′

**Pri-miR160a**

5′-GCTCCCTGTATGCCATATGCT-3′/5′-ATGGCTCCTCATACGCCATC-3′

**Pri-miR166a**

5′-AGGACTCTGGCTCGCTCTAT-3′/5′-GGAATGAAGCCTGGTCCGAA-3′

**Pri-miR164a**

5′-GCACGTGCAAACCAACAAAC-3′/5′-TGGAGAAGTTAAGTACGTGCAA-3′

**MYB33 (AT5G06100)**

5′-CACTCCATTGGTGGTGTGGA-3′/5′-TCGGCTGTGATCAAAAGGCT-3′

**AP2 (AT4G36920)**

5′-CGGCTCAGGATGAACCAACA-3-CGGCCGCTACCAATGTTGCTGCTG-3C

**ARF17 (AT1G77850)**

5RFAGCAAAGAGTCCAGCGACAA-3CAAAGCCTTGGGAGCTAGAACCTGC-3T

**REV (AT5G60690)**

5EVTCACTTGGAAGCGACGACTC-3ACTTGCTAGACCAGCTTGGTTCGCA-3A

**CUC1 (AT3G15170)**

5UCTCTGCTGCTCATACCGATGC-3TGCTGCCAGAAACTGACCAAACGCC-3A

**DCL1 (AT1G01040)**

5′-CGTTGTTATGCGTTTCGACCTTGC-3′/5′-AACGCTGCGTGAGATACATTTCCTC-3′

**SERRATE (At2g27100)**

5′-TGGCTATGGACCACCCGATAGAAG-3′/5′-CCCAGTCCTGATAGCGACCCATAA-3′

**HYL1 (At1g09700)**

5′-CTCTTCGCCGTGCCCTACTAACCTA-3′/5′-GCATACTCCTGCAACCGACTTTTGA-3′

**AGO1 (AT1G48410)**

5′-TGGAACGGTGAATAATTGGATCTGC-3′/5′-TGGTTCCGGATTAAATGCCATGC-3′

**ACTIN2 (At3g18780)**

5′-GCACCCTGTTCTTCTTACCG-3′/5′-AACCCTCGTAGATTGGCACA-3′
