# Supplementary material for: Cesium Toxicity Alters MicroRNA Processing and AGO1 Expressions in Arabidopsis thaliana
Source: PLoS One. 2015 May 6;10(5):e0125514. doi: 10.1371/journal.pone.0125514 (PMC4422737; doi:10.1371/journal.pone.0125514)
Supplement: Supplementary file 3 [file pone.0125514.s002.docx]

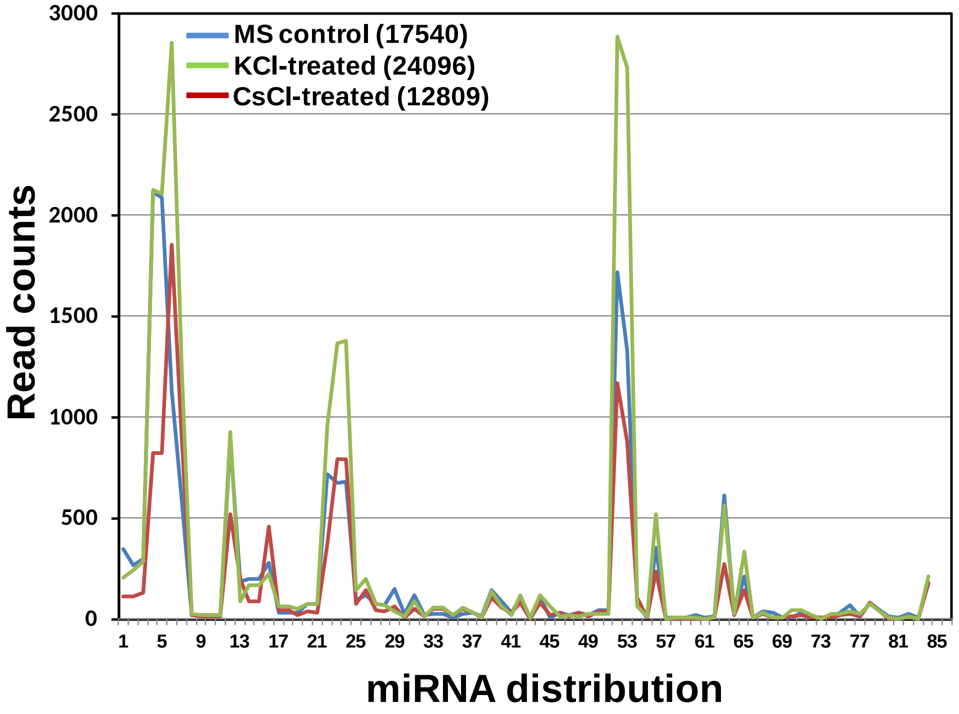
MS control (17540)
KCl-treated (24096)
CsCl-treated (12809)
Read counts
3000
2500
2000
1500
1000
500
0
1
5
9
13
17
21
25
29
33
37
41
45
49
53
57
61
65
69
73
77
81
85
miRNA distribution
