# Supplementary material for: Cesium Toxicity Alters MicroRNA Processing and AGO1 Expressions in Arabidopsis thaliana
Source: PLoS One. 2015 May 6;10(5):e0125514. doi: 10.1371/journal.pone.0125514 (PMC4422737; doi:10.1371/journal.pone.0125514)
Supplement: Supplementary file 4 [file pone.0125514.s003.docx]

A

Down-regulation

miRNA	Log2(TPTM in Salt/MS) CsCl	Log2(TPTM in Salt/MS) KCl
ath-MIR399d	-3.301350472	-1.895131826
ath-MIR399f	-3.181747956	-1.830928707
ath-MIR5026	-2.570054366	-1.789217549
ath-MIR399a	-2.259932147	-2.592908859
ath-MIR838	-2.181012075	-2.059138341
ath-MIR829	-2.010095871	-0.858649022
ath-MIR399b	-1.924762965	-0.979766992
ath-MIR399c	-1.787259441	-0.979766992
ath-MIR170	-1.726213131	-1.191168256
ath-MIR156a-j	-1.654158451	-0.770168102
ath-MIR822	-1.419827335	-0.980905892
ath-MIR158a	-1.356533801	0.009864999
ath-MIR158b	-1.348511505	0.01151229
ath-MIR850	-1.333015169	-1.059138341
ath-MIR169a-n	-1.29412637	-1.925502835
ath-MIR157a-b	-1.279733592	-0.131946607
ath-MIR390a	-1.255012657	-0.215257543
ath-MIR162a	-1.23296267	-0.230432163
ath-MIR162b	-1.231972488	-0.228541984
ath-MIR171a	-1.192354256	-0.481615866
ath-MIR157c-d	-1.172986386	-0.046523959
ath-MIR403	-1.170668524	-0.12006065
ath-MIR846	-1.13947761	-0.806303599
ath-MIR165b	-1.086938311	0.047484454
ath-MIR165a	-1.053847267	0.052311358
ath-MIR781a-b	-1.036622166	-1.499710932
ath-MIR166a-g	-0.957269318	0.411940535
ath-MIR393b	-0.932979833	0.513526205
ath-MIR393a	-0.901675857	-0.367260636
ath-MIR168b	-0.824778459	0.035148465

miRNA	Log2(TPTM in Salt/MS) CsCl	Log2(TPTM in Salt/MS) KCl
ath-MIR168a	-0.819626512	0.050847517
ath-MIR8175	-0.800667294	-0.269315586
ath-MIR400	-0.796514157	-1.279033453
ath-MIR161	-0.773773763	0.068110504
ath-MIR171b-c	-0.754478937	-0.207269853
ath-MIR5642a-b	-0.725958897	-0.428661181
ath-MIR472	-0.688090712	-0.352310295
ath-MIR398a	-0.664558352	0.611677748
ath-MIR319b	-0.654128655	-0.443452289
ath-MIR164c	-0.641000907	0.517557191
ath-MIR1886	-0.635284738	-0.4580261
ath-MIR775	-0.613123088	0.53774285
ath-MIR396b	-0.600153211	1.038756935
ath-MIR8167a-f	-0.571729895	1.153430998
ath-MIR398b-c	-0.568723305	0.557351107
ath-MIR160b	-0.564515209	-0.256511569
ath-MIR408	-0.56238828	0.682941292
ath-MIR396a	-0.554261589	0.749436462
ath-MIR160c	-0.542494033	-0.18335282
ath-MIR160a	-0.528510256	-0.174211973
ath-MIR319a	-0.457166516	-0.022992068
ath-MIR390a	-0.439694623	0.159216784
ath-MIR390b	-0.439694623	0.159216784
ath-MIR773a	-0.434277587	0.5995725
ath-MIR824	-0.430688098	0.105327886
ath-MIR167c	-0.280280112	0.642608999
ath-MIR823	-0.227136756	0.693621606
ath-MIR827	-0.184847332	-0.284562066
ath-MIR447a	-0.157238837	-0.245932089
ath-MIR394a	-0.124859985	-0.860737974
ath-MIR394b	-0.101084233	-0.821915115
ath-MIR393a	-0.060327631	-1.476885127
ath-MIR393b	-0.060327631	-1.476885127

B

Up-regulation

miRNA	Log2(TPTM in Salt/MS) CsCl	Log2(TPTM in Salt/MS) KCl
ath-MIR172c	1.639449117	2.017797706
ath-MIR391	1.251947332	2.787316401
ath-MIR172a	1.043050025	1.172988018
ath-MIR172e	1.024242287	1.154725426
ath-MIR172b	1.020538794	1.157550917
ath-MIR163	0.720380157	-0.332326846
ath-MIR159a	0.711618245	1.336773405
ath-MIR159b	0.607048077	1.080808526
ath-MIR5653	0.603073067	2.661308516
ath-MIR397a-b	0.59263749	-0.123648785
ath-MIR164b	0.441382011	0.970118349
ath-MIR164a	0.429705926	0.953863708
ath-MIR167d	0.269157897	0.718922451
ath-MIR167a	0.222441523	1.011679932
ath-MIR167b	0.218619327	1.010665407
ath-MIR161	0.076077967	-1.077754019
ath-MIR173	0.07353087	0.077938834
ath-MIR159c	0.051495584	0.521577425
ath-MIR858a-b	0.047726277	0.240212718
ath-MIR319c	0.044028262	-0.785361834
